# Human *Borrelia miyamotoi* infection in California: Serodiagnosis is complicated by multiple endemic *Borrelia* species

**DOI:** 10.1371/journal.pone.0191725

**Published:** 2018-02-08

**Authors:** Peter J. Krause, Madeleine Carroll, Natalia Fedorova, Janna Brancato, Cecilia Dumouchel, Fredua Akosa, Sukanya Narasimhan, Erol Fikrig, Robert S. Lane

**Affiliations:** 1 Department of Epidemiology of Infectious Diseases, Yale School of Public Health, New Haven, CT, United States of America; 2 Department of Infectious Diseases, Yale School of Medicine, New Haven, CT, United States of America; 3 Department of Environmental Science, Policy and Management, University of California, Berkeley, CA, United States of America; University of Kentucky College of Medicine, UNITED STATES

## Abstract

To determine whether human *Borrelia miyamotoi* infection occurs in the far-western United States, we tested archived sera from northwestern California residents for antibodies to this emerging relapsing fever spirochete. These residents frequently were exposed to *I*. *pacificus* ticks in a region where *B*. *miyamotoi* tick infection has been reported. We used a two-step *B*. *miyamotoi* rGlpQ assay and a *B*. *miyamotoi* whole-cell lysate (WCL) assay to detect *B*. *miyamotoi* antibody. We also employed *Borrelia hermsii* and *Borrelia burgdorferi* WCL assays to examine if these *Borrelia* induce cross reacting antibody to *B*. *miyamotoi*. Sera were collected from 101 residents in each of two consecutive years. The sera of 12 and 14 residents in years one and two, respectively, were *B*. *miyamotoi* rGlpQ seroreactive. Sufficient sera were available to test 15 of the 26 seropositive samples using *B*. *miyamotoi* and *B*. *hermsii* WCL assays. Two residents in year one and seven residents in year two were seroreactive to both *Borrelia* antigens. Although discernible differences in seroreactivity were evident between the *B*. *miyamotoi* and *B*. *hermsii* WCL assays, infection with one or the other could not be determined with certainty. Sera from two *Borrelia burgdorferi* /*B*. *miyamotoi* seropositive subjects reacted strongly against *B*. *miyamotoi* and *B*. *hermsii* WCL antigens. Ecological, epidemiological, and clinical data implicated *B*. *miyamotoi* as the probable cause of infection among those whose sera reacted against both antigens. Our findings suggest that human *B*. *miyamotoi* infection occurs in northern California and that *B*. *hermsii* and *B*. *burgdorferi* infections produce antibodies that cross-react with *B*. *miyamotoi* antigens. Health care professionals in the far-western United States should be aware that *B*. *miyamotoi* disease may occur throughout the geographic distribution of *I*. *pacificus* and that improved relapsing fever group spirochete antibody assays are urgently needed.

## Introduction

*Borrelia miyamotoi* is a relapsing fever-group spirochete that was discovered in *Ixodes persulcatus* ticks in Japan more than 20 years ago and later determined to cause clinical illness in humans [1–9]. This spirochete can cause a febrile viral-like illness that relapses in up to 10% of patients [2, 5–6]. Immunocompromised patients may experience meningoencephalitis [3, 7–8]. *B*. *miyamotoi* is widespread in the United States in *Ixodes scapularis* ticks in the Northeast and upper Midwest and in *Ixodes pacificus* ticks in the Far West [10–16]. Human cases of relapsing fever due to *B*. *miyamotoi* (hard tick-borne relapsing fever) have been described in the Northeast and upper Midwest, as well as in Russia, the Netherlands, Germany, and Japan [2–9]. In the Northeast, *B*. *miyamotoi* seroprevalence is estimated to be approximately 1 to 3%, which is about one-tenth to one-third that of Lyme disease [4, 17]. No human cases of *B*. *miyamotoi* previously have been reported from the western United States even though *I*. *pacificus* ticks in northern California have a spirochete-infection prevalence similar to or exceeding that of *I*. *scapularis* ticks in the Northeast and upper Midwest [11–16].

To determine whether human *B*. *miyamotoi* infection occurs in the far-western United States, we used a two-step *B*. *miyamotoi* rGlpQ antigen-based antibody assay to test archived sera from residents of a small rural community (population ~150) in northern California. This particular community was located in ecologically diverse Mendocino County, a region where *B*. *miyamotoi* and *Borrelia burgdorferi*-infected *I*. *pacificus* ticks repeatedly have been found [Massachusetts General Hospital Tick-borne Diseases Conference; June 17–20, 2016, Boston, Massachusetts, USA] [11, 14–16, 18–19]. Seroprevalence determination with the *B*. *miyamotoi* GlpQ assay is not affected by Lyme disease infection because *B*. *burgdorferi* does not produce GlpQ antigen, however, several soft tick-borne relapsing fever *Borrelia* species that are endemic in the western United States do produce GlpQ and thus might elicit cross-reacting antibodies against rGlpQ or other *B*. *miyamotoi* antigens [20]. We therefore tested the same archived sera for antibodies against the relapsing fever spirochetes *Borrelia hermsii* and *B*. *miyamotoi* in whole cell lysate (WCL) assays.

## Materials and methods

### Human study population

Serum samples were obtained in 1988 and 1989 from 101 residents of a community at high risk for Lyme disease (CHR) located in the Ukiah area of southern Mendocino County [18–19]. These sera previously were tested for *B*. *burgdorferi* seroreactivity as part of an inter- and intra-laboratory comparative study [18]. Since then, the sera were maintained at -80°C, although they were frozen and thawed a few times prior to *B*. *miyamotoi* testing. In the initial serosurvey [19], subjects were asked to recall any previous Lyme disease diagnosis, history of tick bite in the previous two years, and signs or symptoms suggestive of Lyme disease. The study was carried out with the approval of the Committee for Protection of Human Subjects at the University of California, Berkeley.

### *Borrelia miyamotoi* rGlpQ ELISA and Western blot assays

Serum samples were diluted 1:320 and tested for IgG antibodies against recombinant *B*. *miyamotoi* glycerophosphodiester phosphodiesterase antigen (rGlpQ) using an IgG ELISA [17]. As a negative control for each ELISA plate, we used sera from three healthy participants who had no history of tick bite or tick-borne disease but who lived in an area where Lyme disease is endemic. A signal ≥3 SD above the mean of three non-infected serum controls was considered positive for *B*. *miyamotoi* antibody. As positive controls, we used sera from patients who were confirmed by PCR or serology to have *B*. *miyamotoi* infection.

Serum samples that were equivocal or positive by ELISA were retested for antibodies against rGlpQ using a Western blot IgG antibody assay [17]. Nitrocellulose membrane strips were individually incubated with human serum at a 1:250 dilution. Samples with a 39-kDa band were counted as Western blot positive. Serum samples were considered *B*. *miyamotoi* seropositive if both ELISA IgG and Western blot IgG tests yielded positive results.

### *Borrelia miyamotoi* and *Borrelia hermsii* WCL Western blot antibody assays

Serum samples diluted 1:100 were assayed for antibodies against *B*. *miyamotoi* and *B*. *hermsii* WCL antigens in Western blot assays [21]. For both the *B*. *miyamotoi* and *B*. *hermsii* set of blots, we included serum from a subject who resides in a non-Lyme disease endemic area and had no history of prior *B*. *miyamotoi* infection (negative control). For both sets of blots, we also included serum from a subject who had PCR-confirmed *B*. *miyamotoi* infection (positive control) and from another subject who had confirmed *B*. *hermsii* infection (positive control). Two of us (PJK and SN) independently counted the number of bands for the positive and negative control sera and the CHR sera that were tested using *B*. *miyamotoi* and *B*. *hermsii* whole lysate assays. A seronegative serum was defined as one with the same or fewer bands as the negative control sera. A seroreactive serum was defined as one that had one or more bands than the negative control. Seroreactive sera were further classified into “moderately reactive” if they had one or more bands than the negative control but fewer bands than the positive control or “strongly reactive” if they had at least as many bands as the positive control.

### *Borrelia burgdorferi* WCL Western blot antibody assay

Serum samples from patients with physician diagnosed Lyme disease (erythema migrans rash and *B*. *burgdorferi* seropositive) and no history of *B*. *miyamotoi* infection, and the same *B*. *miyamotoi* seropositive and seronegative controls as described above were diluted 1:100 and assayed for antibodies against *B*. *miyamotoi* and *B*. *burgdorferi*. We used WCL antigens prepared from *in vitro* cultivated *B*. *miyamotoi* or *B*. *burgdorferi* in separate Western blot assays [22–23].

## Results

### Seroreactivity to *B*. *miyamotoi* GlpQ antigen

Clinical and laboratory data were evaluated among 101 of the original 119 CHR subjects whose archived serum samples were available for *B*. *miyamotoi* serologic testing. The mean age of the 101 subjects was 32 years (range 4 to 52 years) and participants included similar percentages of females (51%) and males (49%). Using the two-step ELISA-Western blot rGlpQ assay, we found that sera from 12 subjects in 1988 and 14 subjects in 1989 reacted against *B*. *miyamotoi* rGlpQ antigen (Table 1 and S1 Table).

**Table 1:** *B*. *miyamotoi* rGlpQ ELISA and Western blot test results among CHR subjects.

Study subject	ELISA optical density	ELISA optical density value > positive control cutoff	rGlpQ Western blot
CHR-23 88	0.147	0.037*	POSITIVE
CHR-24 88	0.141	0.031	POSITIVE
CHR-39 88	0.078	0.001	POSITIVE
CHR-40 88	0.082	0.005	POSITIVE
CHR-47 88	0.107	0.030	POSITIVE
CHR-50 88	0.082	0.005	POSITIVE
CHR-52 88	0.087	0.010	POSITIVE
CHR-58 88	0.087	0.010	POSITIVE
CHR-64 88	0.112	0.045	POSITIVE
CHR-68 88	0.093	0.026	POSITIVE
CHR-81 88	0.092	0.025	POSITIVE
CHR-84 88	0.175	0.041	POSITIVE
CHR-26 88	0.059	negative	negative
CHR-45 88	0.093	0.016	negative
CHR-60 88	0.057	negative	negative
CHR-78 88	0.059	negative	negative
CHR-23 89	0.175	0.065	POSITIVE
CHR-26 89	0.080	0.013	POSITIVE
CHR-39 89	0.133	0.056	POSITIVE
CHR-45 89	0.090	0.013	POSITIVE
CHR-47 89	0.143	0.066	POSITIVE
CHR-58 89	0.101	0.034	POSITIVE
CHR-59 89	0.082	0.015	POSITIVE
CHR-60 89	0.123	0.056	POSITIVE
CHR-65 89	0.130	0.063	POSITIVE
CHR-69 89	0.095	0.028	POSITIVE
CHR-78 89	0.080	0.013	POSITIVE
CHR-92 89	0.176	0.060	POSITIVE
CHR-112 89	0.125	0.019	POSITIVE
CHR-116 89	0.109	0.003	POSITIVE

*The negative control values used in the test run for CHR-23 88 were 0.085, 0.065, 0.060. The ≥3 SD cutoff value above the mean of the negative control values was 0.110 (positive cutoff). The optical density reading for CHR-23 88 was 0.147. Thus, the CHR-23 88 value was 0.037 above the positive cutoff value.

### Seroreactivity to *B*. *miyamotoi* and *B*. *hermsii* WCL antigen

**Fig 1:**
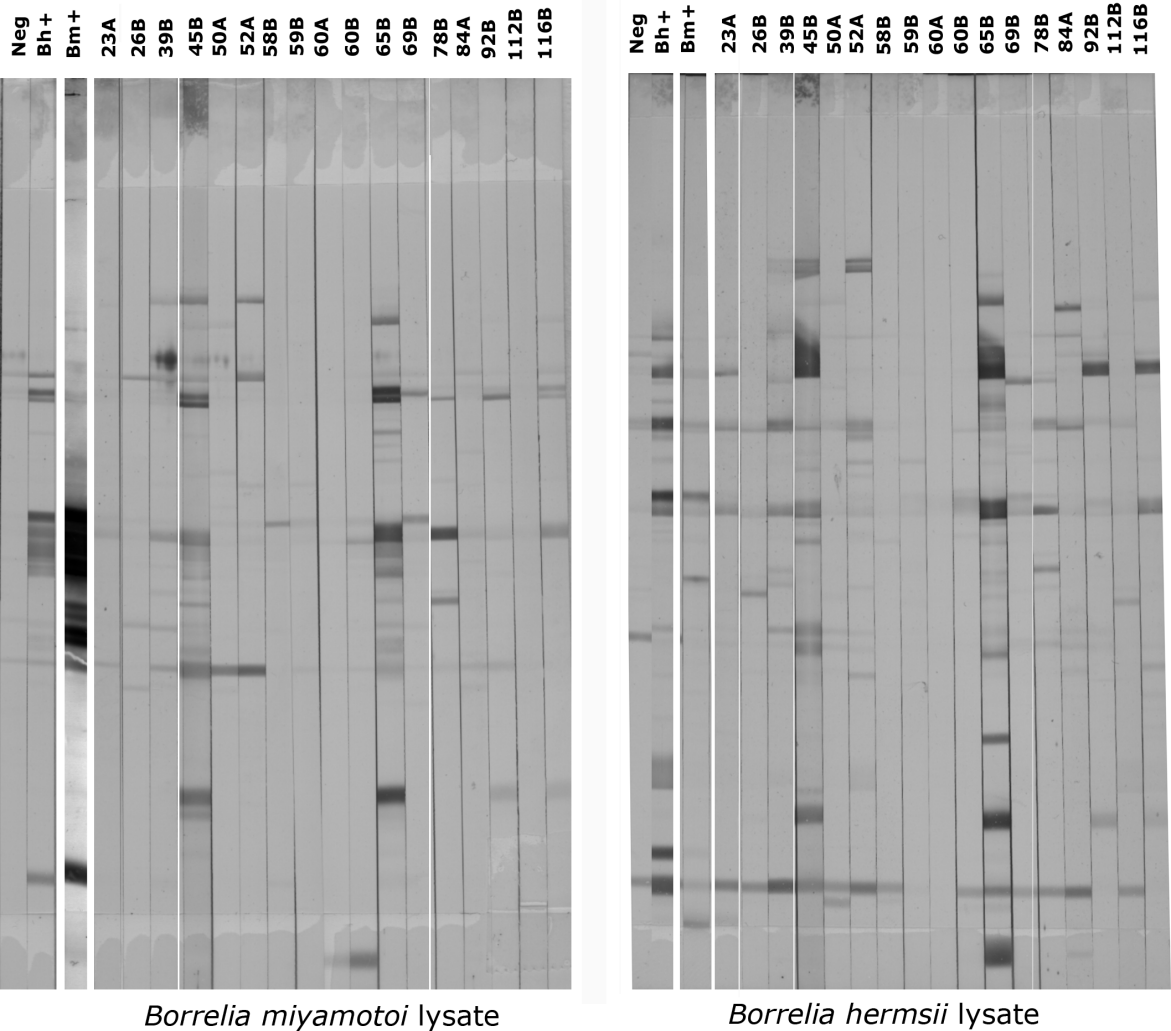
*B*. *miyamotoi* and *B*. *hermsii* WCL antigen Western blot reactivity in serum samples from California subjects whose sera reacted against *B*. *miyamotoi* rGlpQ antigen. Numbers at the top of the rows are subject numbers. For both the *B*. *miyamotoi* and *B*. *hermsii* sets of blots, lane 1 displays the test result for a subject who resided in a non-Lyme disease endemic area and had no history of prior *B*. *miyamotoi* infection (negative control). The blots in lane 2 display the test results for a subject who had confirmed *B*. *hermsii* infection (positive control). The blot in lane 3 displays the test results for a subject who had PCR-confirmed *B*. *miyamotoi* infection (positive control). The positive and negative control sera are the same as those described in Table 2. The other blots display the test results for 15 of 26 *B*. *miyamotoi* GlpQ seropositive CHR subjects who had a sufficient volume of serum remaining to perform *B*. *miyamotoi* and *B*. *hermsii* WCL antibody testing.

A sufficient volume of sera was available from 15 of 26 *B*. *miyamotoi* GlpQ seroreactive subjects for *B*. *miyamotoi* and *B*. *hermsii* WCL antibody testing. Although discernible differences in seroreactivity against *B*. *miyamotoi* and *B*. *hermsii* WCL were noted between the CHR study subjects, infection with one or the other of them could not be distinguished with certainty (Fig 1). Two coauthor analysts (PJK and SN) independently assessed the number of bands on the Western blots to determine bands that represented specific reactivity compared to the negative control sera. They scored the specimens similarly (Table 2). Two of four *B*. *miyamotoi* GlpQ antibody positive subjects and seven of 11 *B*. *miyamotoi* GlpQ antibody positive subjects were seroreactive against *B*. *miyamotoi* and *B*. *hermsii* WCL antigens in 1988 and 1989, respectively.

**Table 2:** *B*. *miyamotoi* and *B*. *hermsii* WCL seroreactivity in CHR subjects who were *B*. *miyamotoi* rGlpQ seroreactive.

Subject	*B*. *miyamotoi* WCL	*B*. *hermsii* WCL
*1988*		
CHR-52	Seroreactive (moderate)^a^	Seroreactive (moderate)
CHR-84	Seroreactive (moderate)	Seroreactive (moderate)
CHR-23	*Seronegative*	*Seronegative*
CHR-50	*Seronegative*	*Seronegative*
*1989*		
CHR-26	Seroreactive (moderate)	Seroreactive (moderate)
CHR-39	Seroreactive (moderate)	Seroreactive (moderate)
CHR-45	Seroreactive (strong) ^b^	Seroreactive (moderate) ^b^
CHR-65	Seroreactive (strong) ^b^	Seroreactive (strong) ^b^
CHR-69	Seroreactive (moderate)	Seroreactive (moderate)
CHR-78	Seroreactive (moderate)	Seroreactive (moderate)
CHR-116	Seroreactive (moderate)	Seroreactive (moderate)
CHR-58	*Seronegative*	*Seronegative*
CHR-59	*Seronegative*	*Seronegative*
CHR-60	*Seronegative*	*Seronegative*
CHR-112	*Seronegative*	*Seronegative*

a Determination of seroreactivity based on the number of bands on the Western blot by two independent analysts. See Results above for definition of strong, moderate and negative reactivity.

b These sera were also *B*. *burgdorferi* seropositive

### Seroreactivity to *B*. *miyamotoi* and *B*. *burgdorferi* WCL antigens

**Fig 2:**
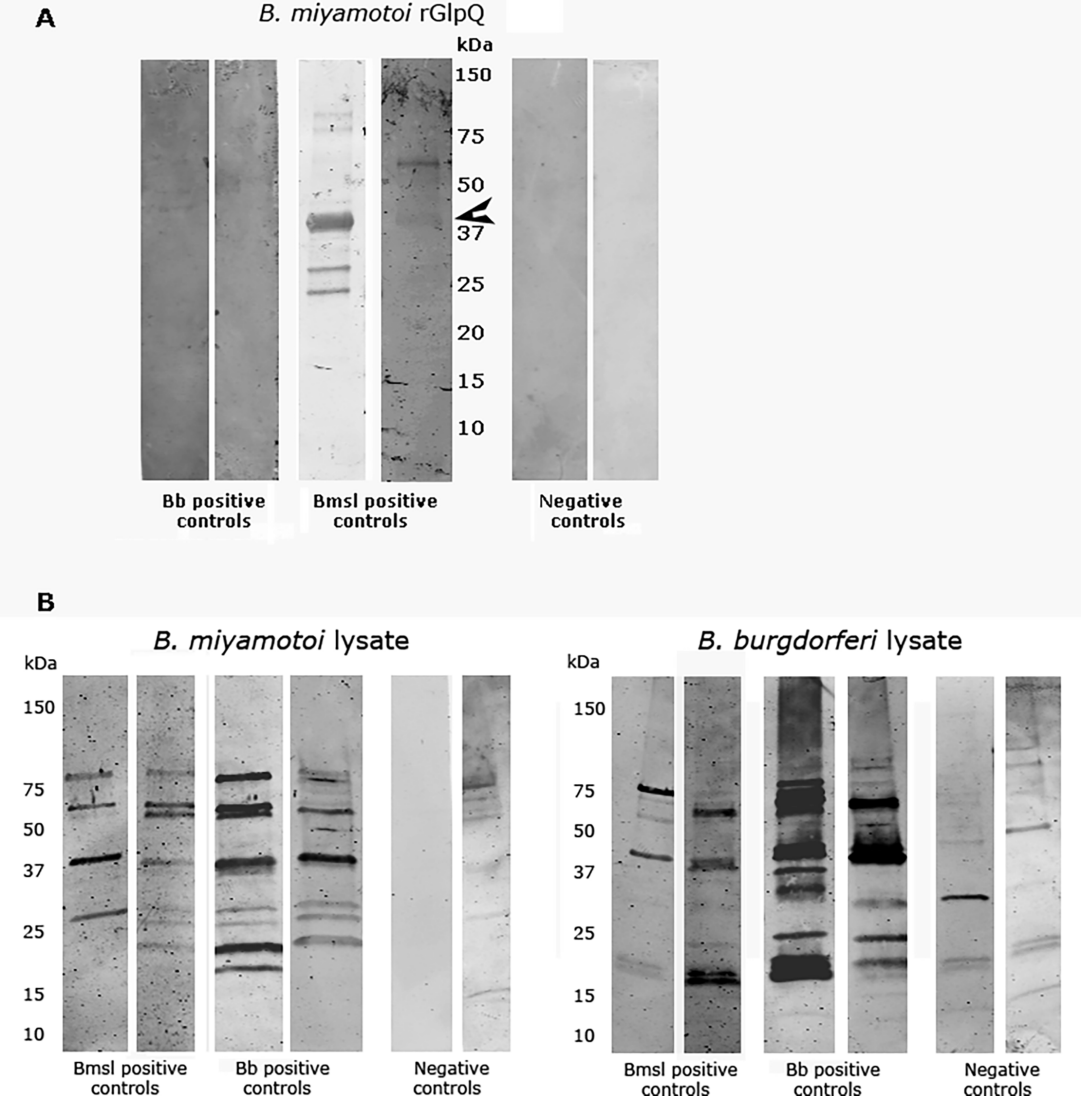
*B*. *burgdorferi* and *B*. *miyamotoi* WCL Western blot reactivity in serum samples from New England residents whose sera were reactive against *B*. *burgdorferi* and *B*. *miyamotoi* antigen. **2A.** *B*. *miyamotoi* rGlpQ Western blot results of sera from patients who previously had *B*. *miyamotoi* sensu lato infection alone (Bmsl positive control subject, PCR confirmed *B*. *miyamotoi* infection) or Lyme disease alone (Bb positive control, erythema migrans rash and *B*. *burgdorferi* seropositive using the standard two-step ELISA and Western blot assay). The arrow indicates the rGlpQ specific band. **2B.** *B*. *miyamotoi* and *B*. *burgdorferi* WCL Western blot results of sera from the same patients as in Fig 2A.

Two CHR residents (CHR 45 and CHR 65) who were seropositive for *B*. *miyamotoi* GlpQ and WCL also were seropositive for *B*. *burgdorferi*. They had coinfection or sequential infection with these two *Borrelia*. Their sera reacted more strongly to *B*. *miyamotoi* WCL antigen than did any of the other CHR *B*. *miyamotoi* seropositive sera (Fig 1). To determine if the strong reaction against *B*. *miyamotoi* WCL antigen among these two *B*. *miyamotoi* and *B*. *burgdorferi*-infected subjects was due in part to *B*. *burgdorferi* cross-reacting antibody, we tested sera from patients who had experienced *B*. *miyamotoi* infection alone (positive *B*. *miyamotoi* control subject), Lyme disease alone (erythema migrans rash and *B*. *burgdorferi* seropositive using the standard ELISA-Western blot assay), and neither infection (negative control) using a *B*. *burgdorferi* WCL assay and a *B*. *miyamotoi* WCL assay using a different *B*. *miyamotoi* WCL assay than that described above. The sera from patients with Lyme disease were reactive against *B*. *burgdorferi* WCL and against several *B*. *miyamotoi* bands in the *B*. *miyamotoi* WCL assay (Fig 2B) but did not react to *B*. *miyamotoi* GlpQ antigen in a Western blot assay (Fig 2A). The sera from patients with *B*. *miyamotoi* were seroreactive against several *B*. *burgdorferi* bands in the *B*. *burgdorferi* WCL assay (Fig 2B).

### Ecological and epidemiological evidence of *B*. *miyamotoi* infection

Ecological and epidemiological evidence indicates that CHR subjects whose sera reacted against *B*. *miyamotoi* GlpQ and WCL antigens and *B*. *hermsii* WCL antigen were much more likely to have been infected with *B*. *miyamotoi* than either *B*. *hermsii* or *Borrelia parkeri*, two other relapsing fever spirochetes present in northern California. *B*. *miyamotoi* has been detected in ticks from Mendocino County but not *B*. *hermsii* or *B*. *parkeri*. Indeed, human cases caused by the latter two spirochetes are acquired distant from the CHR community and the incidence of human infection with them is either low (*B*. *hermsii*) or rare (*B*. *parkeri*) statewide. A high percentage (78%) of CHR residents reported tick bite, which is more consistent with hard-bodied than soft-bodied ticks. The latter feed for less than an hour at night and are very seldom noticed in people who suffer soft tick-borne relapsing fever. One or more relapsing fever episodes typically occur in patients infected with all soft tick-borne relapsing fever infections but with only 10 percent or fewer people experiencing *B*. *miyamotoi* infection. No relapsing fever episodes were reported by CHR residents in 1988 and 1989.

## Discussion

Our principal aim was to determine whether residents of the far-western United States are at risk of human *B*. *miyamotoi* infection. The CHR residents were an ideal group to screen because *B*. *miyamotoi*-infected *I*. *pacificus* ticks have been identified from nearby areas in Mendocino County. Moreover, the members of this rural community experienced extraordinarily high tick exposure rates that far exceed published exposure rates from the Northeastern United States [19, 24, 25]. It was therefore not surprising that 2 of 101 CHR residents in 1988 and 7 of 101 CHR residents in 1989 were seroreactive to *B*. *miyamotoi* GlpQ and WCL antigens. Although these residents were also seroreactive to *B*. *hermsii* antigens, this was likely a result of *B*. *miyamotoi* cross reacting antibody, rather than previous *B*. *hermsii* infection. Human *B*. *hermsii* infection is infrequently reported in mountainous areas far to the east of Mendocino County and none of the residents reported having experienced relapsing fever episodes that are characteristic of *B*. *hermsii* during the two-year study period. Although we found that *B*. *burgdorferi*, like *B*. *hermsii*, can elicit cross-reacting antibody against *B*. *miyamotoi* antigens, none of our putative *B*. *miyamotoi* seropositive CHR residents could have had Lyme disease alone because they reacted against *B*. *miyamotoi* GlpQ antigen and *B*. *burgdorferi* does not produce GlpQ [20]. In sum, our data suggests that human *B*. *miyamotoi* infection occurs in the Far West and that *B*. *miyamotoi*, *B*. *hermsii* and *B*. *burgdorferi* spirochetes share many proteins that elicit cross reacting antibodies, which complicate the serodiagnosis of *B*. *miyamotoi* and other relapsing fever infections.

Several ecological and epidemiological observations support the possibility of human *B*. *miyamotoi* infection in northern California. We found that between 2 and 7 of the 101 CHR residents were seroreactive against *B*. *miyamotoi* GlpQ and WCL antigen in 1988 and 1989, respectively. This seropostitivity range approximates the 0.5% to 15% *B*. *miyamotoi* infection range that has been found in *I*. *pacificus* ticks in Northern California [11, 14, 15]. The CHR residents built their homes on a former cattle ranch during the 1970s where *I*. *pacificus* and its manifold vertebrate and reservoir hosts abound [19]. Over three quarters (78%) of them reported tick-bites 1–2 years prior to enrollment and in a follow-up study, 79% had elevated anti-*I*. *pacificus* saliva antibodies [24]. By comparison, these tick-exposure rates are nearly triple the tick-bite incidence reported for residents of Block Island, Rhode Island (29%) where Lyme disease and babesiosis are highly endemic [25, 26]. Other ecological factors likely contributing to the force of *B*. *miyamotoi* transmission to humans at the CHR include the prolonged activity periods of *I*. *pacificus* nymphs and adults (compared to the lesser year-round activity periods of *I*. *scapularis* in the Northeast) that collectively span most of the year, as well as the occurrence of transovarial transmission of *B*. *miyamotoi* in its primary *Ixodes* spp. tick vectors [27, 28]. It is not likely that CHR residents were infected with either *B*. *hermsii* or *B*. *parkeri*. Neither of these two soft tick-borne relapsing fever species have been detected in ticks from Mendocino County [11, 14, 15]. *B*. *hermsii* infection incidence averages only 5 to 7 reported cases per year in California, and it occurs mainly in high mountains located about 500 to 1000 km to the east of the CHR [29, 30]. *B*. *hermsii* typically causes 1–10 febrile episodes accompanied by a viral-like illness that tends to occur in clusters among family members or friends who frequent the same mountain cabins. None of the CHR residents reported a previous diagnosis of soft tick-borne relapsing fever or episodes of a relapsing fever-like illness 1–2 years pre-entry or 1 year post-entry. *B*. *parkeri*, was incriminated in only a single human case in the distant Central Valley during the1940’s [31]. Human cases have not been reported due to a third soft tick-borne relapsing spirochete, *B*. *coriaceae*, first isolated from the soft tick *Ornithodoros coriaceus* in southern Mendocino County decades ago [32].

Although we found that *B*. *burgdorferi* also can elicit cross-reacting antibody against *B*. *miyamotoi* antigens, none of our putative *B*. *miyamotoi* seropositive CHR residents could have had Lyme disease alone because they reacted against *B*. *miyamotoi* GlpQ antigen and *B*. *burgdorferi* does not produce GlpQ [20]. Interestingly, the strongest reactions against *B*. *miyamotoi* WCL antigen of all the *B*. *miyamotoi/ B*. *hermsii* seropositive CHR sera were in the two residents who also reported previous Lyme disease. The intense reactivity to *B*. *miyamotoi* and *B*. *hermsii* WCL antigens is likely due in part to *B*. *burgdorferi* cross-reacting antibody [33]. Recent studies have shown that sera from *B*. *miyamotoi*-infected patients cross react to *B*. *burgdorferi* C6 peptide [16, 34]. Cross reacting antibody against *B*. *miyamotoi/B*. *herrmsii* WCL antigens also may have resulted from previous *Haemophilus influenza* or certain other gram-negative bacterial infections that produce GlpQ. The genetic difference between the GlpQ of these pathogens and *B*. *miyamotoi* is so wide, however, that significant cross-reactivity is unlikely [17, 20].

Our study was subject to several limitations. We enrolled a relatively small number of subjects and controls. No PCR-amplifiable *B*. *miyamotoi* DNA was detected in sera from CHR subjects, although these samples were obtained a few weeks to months after acute illness was likely to have occurred. The positive control sera used for the *B*. *miyamotoi* set of blots in Fig 1 was intensely positive and a number of bands likely bled into one another. We counted this central confluence as a single band but this had no effect on the number of seroreactive subjects as they were defined based on comparison with the number of bands in the negative control sera. *B*. *burgdorferi* antibody testing of the CHR residents was carried out before the development of the current two-tiered assay but the IFA assay that was used was determined to be highly sensitive and specific [18, 35]. Finally, archived sera had been frozen and thawed a few times before *B*. *miyamotoi* and *B*. *hermsii* antibody testing, but this is unlikely to have reduced antibody concentration.

## Conclusions

Taken together, our serological, ecological and epidemiological data provide presumptive evidence that *B*. *miyamotoi* occasionally infects residents of Mendocino County in northwestern California. The presence of two soft-tick borne borreliae in California that either occasionally (*B*. *hermsii*) or rarely (*B*. *parkeri*) infect people in other counties, plus the occurrence of a third species (*B*. *coriaceae*) of uncertain human significance, precludes absolute confirmation that the seroreactive CHR residents were infected with *B*. *miyamotoi*. Our findings also highlight the pressing need to develop a serodiagnostic assay capable of differentiating all members of the relapsing fever group.

## Supporting information

S1 Table *B*. *miyamotoi* rGlpQ ELISA- Western blot results in CHR subjects, 1988–1989. (DOCX)
