# Supplementary material for: Human Borrelia miyamotoi infection in California: Serodiagnosis is complicated by multiple endemic Borrelia species
Source: PLoS One. 2018 Feb 8;13(2):e0191725. doi: 10.1371/journal.pone.0191725 (PMC5805228; doi:10.1371/journal.pone.0191725)
Supplement: S1 Table — (DOCX) [file pone.0191725.s001.docx]

**S1 Table. *B. miyamotoi* rGlpQ ELISA- Western blot results in CHR subjects, 1988-1989**

| **Sample ID** | ***B. miyamotoi* rGlpQ ELISA-WB** | |
| --- | --- | --- |
|  | **1988** | **1989** |
| CHR-1 89 | N/A* | negative |
| CHR-2 88 | negative | N/A |
| CHR-4 88 | negative | N/A |
| CHR-5 88 | negative | N/A |
| CHR-6 88 | negative | N/A |
| CHR-7 89 | N/A | negative |
| CHR-8 89 | N/A | negative |
| CHR-9 88 | negative | N/A |
| CHR-10 88/CHR-10 89 | negative | negative |
| CHR-11 88/ CHR-11 89 | negative | negative |
| CHR-13 88 / CHR-13 89 | negative | negative |
| CHR-14 89 | N/A | negative |
| CHR-15 88/ CHR-15 89 | negative | negative |
| CHR-16 88/CHR-16 89 | negative | negative |
| CHR-17 88/CHR-17 89 | negative | negative |
| CHR-18 89 | N/A | negative |
| CHR-19 88/CHR-19 89 | negative | negative |
| CHR-20 88/CHR-20 89 | negative | negative |
| CHR-21 88/CHR-21 89 | negative | negative |
| CHR-22 89 | N/A | negative |
| CHR-23 88/CHR-23 89 | POSITIVE | POSITIVE |
| CHR-24 88/CHR-24 89 | POSITIVE | negative |
| CHR-25 88/CHR-25 89 | negative | negative |
| CHR-26 88/CHR-26 89 | negative | POSITIVE |
| CHR-27 89 | N/A | negative |
| CHR-28 88/CHR-28 89 | negative | negative |
| CHR-29 89 | N/A | negative |
| CHR-30 88 | negative | N/A |
| CHR-31 88/CHR-31 89 | negative | negative |
| CHR-32 88/CHR-32 89 | negative | negative |
| CHR-33 88/CHR-33 89 | negative | negative |
| CHR-34 88 | negative | N/A |
| CHR-35 89 | N/A | negative |
| CHR-36 88/CHR-36 89 | negative | negative |
| CHR-37 88/CHR-37 89 | negative | negative |
| CHR-38 88/CHR-38 89 | negative | negative |
| CHR-39 88/CHR-39 89 | POSITIVE | POSITIVE |
| CHR-40 88/CHR-40 89 | POSITIVE | negative |
| CHR-42 89 | N/A | negative |
| CHR-43 88/CHR-43 89 | negative | negative |
| CHR-44 88/CHR-44 89 | negative | negative |
| CHR-45 88/CHR-45 89 | negative | POSITIVE |
| CHR-47 88/CHR-47 89 | POSITIVE | POSITIVE |
| CHR-48 88 | negative | N/A |
| CHR-50 88 | POSITIVE | N/A |
| CHR-51 88/CHR-51 89 | negative | negative |
| CHR-52 88/CHR-52 89 | POSITIVE | negative |
| CHR-53 89 | N/A | negative |
| CHR-54 88 | negative | N/A |
| CHR-55 88 | negative | N/A |
| CHR-56 88 | negative | N/A |
| CHR-57 88 | negative | N/A |
| CHR-58 88/CHR-58 89 | POSITIVE | POSITIVE |
| CHR-59 89 | N/A | POSITIVE |
| CHR-60 88/CHR-60 89 | negative | POSITIVE |
| CHR-61 88 | negative | N/A |
| CHR-63 88 | negative | N/A |
| CHR-64 88 | POSITIVE | N/A |
| CHR-65 89 | N/A | POSITIVE |
| CHR-68 88 | POSITIVE | N/A |
| CHR-69 89 | N/A | POSITIVE |
| CHR-72 88/CHR-72 89 | negative | negative |
| CHR-73 88/CHR-73 89 | negative | negative |
| CHR-74 88/CHR-74 89 | negative | negative |
| CHR-75 89 | N/A | negative |
| CHR-76 88 | negative | N/A |
| CHR-77 89 | N/A | negative |
| CHR-78 88/CHR-78 89 | negative | POSITIVE |
| CHR-80 88 | negative | N/A |
| CHR-81 88 | POSITIVE | N/A |
| CHR-82 88/CHR-82 89 | negative | negative |
| CHR-83 88 | negative | N/A |
| CHR-84 88/CHR-84 89 | POSITIVE | negative |
| CHR-85 88 | negative | N/A |
| CHR-86 88 | negative | N/A |
| CHR-87 88 | negative | N/A |
| CHR-88 88 | negative | N/A |
| CHR-89 88 | negative | N/A |
| CHR-90 89 | N/A | negative |
| CHR-91 89 | N/A | negative |
| CHR-92 89 | N/A | POSITIVE |
| CHR-93 88/CHR-93 89 | negative | negative |
| CHR-94 88 | negative | N/A |
| CHR-95 89 | N/A | negative |
| CHR-96 88/CHR-96 89 | negative | negative |
| CHR-98 88/CHR-98 89 | negative | negative |
| CHR-99 88/CHR-99 89 | negative | negative |
| CHR-101 89 | N/A | negative |
| CHR-102 88 | negative | N/A |
| CHR-103 88/CHR-103 89 | negative | negative |
| CHR-104 88/CHR-104 89 | negative | negative |
| CHR-105 88 | negative | N/A |
| CHR-106 88 | negative | N/A |
| CHR-107 88/CHR-107 89 | negative | negative |
| CHR-108 88/CHR-108 89 | negative | negative |
| CHR-109 88/CHR-109 89 | negative | negative |
| CHR-112 89 | N/A | POSITIVE |
| CHR-114 88/CHR-114 89 | negative | negative |
| CHR-116 89 | N/A | POSITIVE |
| CHR-118 89 | N/A | negative |
| CHR-119 89 | N/A | negative |
|  |  |  |
| * Not available |  |  |
